# The Histidine-Phosphocarrier Protein of the Phosphoenolpyruvate: Sugar Phosphotransferase System of *Bacillus sphaericus* Self-Associates

**DOI:** 10.1371/journal.pone.0069307

**Published:** 2013-07-26

**Authors:** Rosa Doménech, José G. Hernández-Cifre, Julio Bacarizo, Ana I. Díez-Peña, Sergio Martínez-Rodríguez, Claudio N. Cavasotto, José García de la Torre, Ana Cámara-Artigás, Adrián Velázquez-Campoy, José L. Neira

**Affiliations:** 1 Instituto de Biología Molecular y Celular, Universidad Miguel Hernández, Elche (Alicante), Spain; 2 Departamento de Química Física, Universidad de Murcia, Murcia, Spain; 3 Departamento de Química y Física, Campus de Excelencia Internacional Agroalimentario, Universidad de Almería, Almería, Spain; 4 Instituto de Investigación en Biomedicina de Buenos Aires (IBioBA)-CONICET- Partner Institute of the Max Planck Society, Buenos Aires, Argentina; 5 Instituto de Biocomputación y Física de Sistemas Complejos, Unidad Asociada IQFR-CSIC-BIFI, Universidad de Zaragoza, Zaragoza, Spain; 6 Fundación ARAID, Diputación General de Aragón, Zaragoza, Spain; 7 Departamento de Bioquímica y Biología Molecular y Celular, Universidad de Zaragoza, Zaragoza, Spain; Russian Academy of Sciences, Institute for Biological Instrumentation, Russian Federation

## Abstract

The phosphotransferase system (PTS) is involved in the use of carbon sources in bacteria. *Bacillus sphaericus*, a bacterium with the ability to produce insecticidal proteins, is unable to use hexoses and pentoses as the sole carbon source, but it has *ptsHI* genes encoding the two general proteins of the PTS: enzyme I (EI) and the histidine phosphocarrier (HPr). In this work, we describe the biophysical and structural properties of HPr from *B. sphaericus*, HPr^bs^, and its affinity towards EI of other species to find out whether there is inter-species binding. Conversely to what happens to other members of the HPr family, HPr^bs^ forms several self-associated species. The conformational stability of the protein is low, and it unfolds irreversibly during heating. The protein binds to the N-terminal domain of EI from *Streptomyces coelicolor*, EIN^sc^, with a higher affinity than that of the natural partner of EIN^sc^, HPr^sc^. Modelling of the complex between EIN^sc^ and HPr^bs^ suggests that binding occurs similarly to that observed in other HPr species. We discuss the functional implications of the oligomeric states of HPr^bs^ for the glycolytic activity of *B. sphaericus*, as well as a strategy to inhibit binding between HPr^sc^ and EIN^sc^.

## Introduction

The bacterial phosphoenolpyruvate (PEP): sugar phosphotransferase system (PTS) regulates the use of carbon sources in bacteria. It is involved in: (i) cell movement towards carbon sources (chemotaxis); (ii) transport and uptake of several carbohydrates through the cell wall; and, (iii) regulation of several metabolic pathways in both gram-negative and gram-positive bacteria [1]–[4]. The basic composition of the PTS is similar in all species described so far; it is formed by a cascade of phosphoryl-transfer steps from PEP to the sugar-specific enzyme II permeases (EIIs). The first two proteins in the cascade are common to all PTS substrates: the phosphocarrier proteins enzyme I and HPr. In the first step of the PTS, EI is phosphorylated by PEP at the active-site histidine; subsequently, the phosphoryl group is transferred to HPr, at its active-site histidine [2], [5], [6]; HPr transfers the phosphate to the active site of an EII permease (usually, but not exclusively, at a histidine residue). In low-G+C gram-positive bacteria, and a few gram-negative organisms, HPr can be also phosphorylated by an ATP-dependent kinase on a serine residue.

*Bacillus sphaericus* is a low-G+C gram-positive bacterium comprising aerobic and mesophilic bacilli, which forms spherical endospores [7]. Some of the strains can synthesize insecticidal proteins active against mosquito species of the genera *Anopheles*. These bacteria metabolize a wide variety of amino acids and organic compounds, but they cannot use hexoses and pentoses as the sole carbon source [8]; thus, alternative media based on starch or molasses are used in these bacteria for toxin production. The inability to metabolize those carbohydrates by *B. sphaericus* has been attributed to the failure to transport either glucose or sucrose [9]. Although the bacterium does not use hexoses or pentoses as a carbon source, it has the *ptsHI* genes encoding the HPr and EI proteins [10], and it shows an enzymatic activity in other glycolytic pathways [11]. Interestingly enough, the HPr shows a low sequence similarity with other members of the family. Therefore, we were first interested in finding out whether the structure of the HPr^bs^ was similar to that of other members of the family, and second, whether its conformational stability was identical to those of the other HPrs.

*Streptomyces* is a soil-dwelling gram-positive actinomycete, with a high-G+C content, that grows on a variety of carbon sources. The complete genome of *S. coelicolor* has been sequenced, and the different components of the PTS have been reported [12]–[14]. We have undertaken an extensive description of the structures and conformational stabilities of HPr^sc^ and EI^sc^, as a first step to understand their binding reaction [15]–[18], and to design potential inhibitors against the PTS in *S. coelicolor*, which could be used as new general antibiotics against bacteria. We have also determined the affinities of the N terminus of wild-type EI, EIN^sc^, for HPr^sc^ [19], and of HPr^sc^ for several peptides derived from EIN^sc^, containing the active-site region or nearby polypeptide patches [20].

In this work, we determined: (i) the biophysical features of HPr^bs^; (ii) the binding of HPr^bs^ to wild-type EIN^sc^, and to its phosphomimetic mutant at the active-site histidine (EINH186D); (iii) the binding to the fragments derived from EIN^sc^, containing the active site and nearby regions; and, (iv) the modeled structure of the EIN^sc^:HPr^bs^ complex. The aim of this work is two-fold. First, to find new inhibitors of the EIN^sc^:HPr^sc^ complex formation; and second to describe the conformational features of HPr^bs^ to allow for a comparison with the properties of other HPrs. To address both questions, we used an array of biophysical techniques and theoretical methods. Our results show that, conversely to what happens with other HPr members, HPr^bs^ forms oligomers; furthermore, the protein has a very low stability. Modelled structures suggest that the protein binds to EIN^sc^ similarly as other complexes in the family do [21]; however, the ITC data indicate that the binding affinity of EIN^sc^ for HPr^bs^ is higher than that for its own species (HPr^sc^), suggesting that oligomerization of HPr^bs^ alters the recognition process. We discuss the importance of this new self-associated state and the low stability of the protein in the HPr family.

## Materials and Methods

### (a) Materials

The Ni^2+^ resin was from GE Healthcare (Barcelona, Spain). Dialysis tubing (Spectra Por), with a molecular weight cut-off of 3500 Da, was from Spectrum Laboratories (Japan). Deuterium oxide was obtained from Apollo Scientific (Stockport, UK), and sodium trimethylsilyl-[2,2,3,3-^2^H_4_]-propionate was from Sigma (Barcelona, Spain). Trypsin proteomic grade was from Sigma (Barcelona, Spain). Markers were from BioRad or Sigma (low-molecular weight range). Standard suppliers were used for all other chemicals. Water was deionized and purified on a Millipore system.

### (b) Protein expression and purification

The vector (pALE-12) containing the HPr^bs^ was a kind gift by C. Sánchez-Rivas (Universidad de Buenos Aires, Argentina). The protein was cloned into the pQE30 plasmid (Qiagen) [10] and expressed in M15 cells. Cells were grown for 3 hours after induction with 0.8 mM IPTG at 37°C, when the culture suspension optical density at 600 nm was 0.6–0.8.

Cells were harvested by centrifugation in a Beckman Coulter J2-HS (Germany) for 15 min at 6000 g, and frozen at −80°C until they were used. The cell pellet was treated with 50 ml of lysis buffer A (500 mM NaCl, 5 mM imidazole, 20 mM Tris (pH 8.0), 0.1% Triton X-100 and 1 mM β-mercaptoethanol) and one tablet of Complete-EDTA-free protease inhibitor (Roche, Germany). The suspension was sonicated 10 times in ice with bursts of 45 s at the maximum power of the sonicator (Model 102-C, Branson, USA), interleaved with 15-s periods on ice. The resultant solution was centrifuged for 50 min at 27000 g, and the supernatant was separated. SDS-PAGE gels showed that HPr^bs^ was expressed as inclusion bodies. The cellular pellet was washed twice with buffer A, but with no protease inhibitor tablet added. The inclusion bodies were resuspended in buffer B (20 mM Tris buffer (pH 8.0), 500 mM NaCl, 1 mM β-mercaptoethanol and 8 M urea). The suspension was sonicated 10 times in ice with bursts of 45 s at the maximum power of the sonicator, interleaved with 15-s periods on ice. The resultant solution was centrifuged for 1 h at 27000 g.

The supernatant, resulting from the culture of five 2 l-flasks, was added to 5 ml of Ni^2+^ resin. The slurry was kept at 5°C for 1 hour with constant agitation, and then transferred to a column sleeve, where the resin was separated from the supernatant by gravity. The resin was incubated with 20 ml of buffer C (500 mM NaCl, 15 mM imidazole, 20 mM Tris (pH 8.0), 1 mM β-mercaptoethanol and 8 M urea) for 20 minutes. The supernatant was then separated from the resin by gravity. Finally, the protein was eluted by washing the resin with 2×20 ml of buffer D (500 mM NaCl, 300 mM imidazole, 20 mM Tris (pH 8.0), 1 mM β-mercaptoethanol and 8 M urea). Samples containing HPr^bs^ were extensively dialyzed against water; the sample precipitated during dialysis. We spun down the dialyzed sample to separate the precipitate from the supernatant at 20000 g during 1 hour. We did not try to re-dissolve the precipitate.

As a final purification step, the protein was purified by using a gel filtration Superdex 75 16/60 column coupled to an AKTA system (GE Healthcare), following the absorbance at 280 nm, in Tris 50 mM (pH 7.5) and 150 mM NaCl. Previously, the samples were concentrated by using Amicon centrifugal devices of a molecular weight cut-off of 3500 Da; the sample precipitated during concentration. The supernatant was separated from the precipitated fraction within the centrifugal device, and we continued working with the former fraction. These results suggest that the protein has a high tendency to self-associate. The protein eluted from the preparative gel filtration column as a main peak at ∼80 ml, although additional peaks were observed eluting at larger volumes than the bed volume of the column (probably due to protein-column interactions). It is important to note that in the preparative column used to purify the protein, the HPr^bs^ also appeared at the elution volume (∼80 ml) close to that expected for a monomeric protein (as it occurred in the SEC experiments with a high resolution column, see below); this is due to the protein-concentration dilution effect within the column matrix. The protein was extensively dialyzed against water, flash frozen and stored at – 80°C until it was used. The HPr^bs^ concentration was determined from the absorbance of individual amino acids at 280 nm [22], by using a Shimadzu UV-1601 spectrophotometer (Japan), in a 1-cm-path-length cell (Hellma). The sequence of HPr^bs^ only contains a Tyr residue, and there are no Cys residues (Fig. S1 in file S1).

The wild-type EIN^sc^ and mutant EINH186D were expressed and purified as described [19]. The peptides containing the active site and nearby regions of EIN^sc^ are: (i) the so-called EINbsite (FVTEEGGPTSHSAILARA-amidated), containing the active-site histidine of EIN^sc^ (His186); and, (ii) the so-called EINosite (YRALLAGAGEYLAGRVADLDD-amidated), containing a polypeptide region that has contacts with the active-site His of HPr in the EIN^ec^:HPr^ec^ complex [21]. The concentrations of the EIN^sc^ species and those of the peptides were determined from the absorbance of individual amino acids at 280 nm [22]. Peptides were synthesized as described [20].

### (c) Fluorescence

Spectra were collected on a Cary Eclipse spectrofluorometer (Varian, California, USA) interfaced with a Peltier system. A 1-cm path-length quartz cell (Hellma) was used. The excitation and emission slits were always set to 5 nm. Experiments were performed in 50 mM Tris buffer, pH 7.0. Protein concentrations were 15 or 30 μM.

#### (c.1.) Steady-state fluorescence measurements

Intrinsic fluorescence spectra were acquired by excitation at 278 nm, collecting data between 300 and 400 nm.

Spectra in the presence of 100 μM of ANS were acquired by excitation at 380 nm, between 400 and 600 nm.

#### (c.2.) Thermal denaturations

Excitation was carried out at 278 nm, and fluorescence emission was collected at 308 and 315 nm. The scan rate was 60°C h^−1^ and the data were acquired every 0.2°C. Thermal denaturations were irreversible.

#### (c.3.) Chemical denaturations

Chemical denaturations at pH 7.0 and 25°C were performed by dissolving protein samples at different urea concentrations, ranging from 0 to 6 M. The experiments were repeated twice. Reversibility experiments were carried out by dissolving the sample in 8 M urea, and diluting this stock solution until the corresponding denaturant concentration was obtained. Experiments were fully reversible.

### (d) Circular dichroism (CD)

Spectra were collected on a Jasco J810 (Japan) spectropolarimeter fitted with a thermostated cell holder and interfaced with a Peltier unit. The instrument was periodically calibrated with (+) 10-camphorsulphonic acid. Experiments in the far-UV CD were carried out in 0.1-cm path-length cells (Hellma) with a protein concentration of 15 or 30 μM, in 50 mM Tris buffer, pH 7.0.

#### (d.1) Steady-state measurements

Spectra were acquired in the far-UV CD with a response time of 1 s and averaged over six scans, with a scan speed of 50 nm/min. The step resolution was 0.2 nm and the bandwidth was 1 nm. Experiments were also attempted in the near-UV with a 0.5-cm path-length cell, 60 μM of protein, and averaging over eight scans.

Experiments in the far-UV CD were carried out at different pHs and 25°C to study the pH-dependent conformation of the protein. The salts and acids used in buffer preparation were: pH 2.0–3.0, H_3_PO_4_; pH 3.0–4.0, H-COOH; pH 4.0–5.5, CH_3_-COOH; pH 6.0–7.0, NaH_2_PO_4_; pH 7.5–9.0, Tris acid; pH 9.5–11.0, Na_2_CO_3_; pH 11.5–13.0, Na_3_PO_4_. The pH was measured with an ultra-thin Aldrich electrode in a Radiometer (Copenhagen, Denmark) pH-meter.

The titration curves were fitted to the Henderson-Hasselbalch equation:
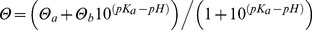
(1)where Θ is the measured ellipticity for a particular pH; Θ_a_ is the ellipticity of the species at low pH; Θ_b_ is the ellipticity of the species at high pH; and p*K*_a_ is the titration midpoint of the pH-transition. Fittings to Eq. 1 were carried out by using the general curve-fit option of Kaleidagraph (Abelbeck software) running on a PC computer.

#### (d.2.) Thermal denaturations

Thermal denaturations were performed at a constant heating rate of 60°C h^−1^, with a response time of 8 s. Thermal scans were collected in the far-UV region at 222 nm from 25 to 95°C, with data collection every 0.2°C. The experiments were repeated twice. Samples were transparent and no precipitation was observed after heating. However, the steady-state spectra of HPr^bs^ after cooling to 25°C were not identical to those obtained before heating. In addition, the monitored voltages of the photomultiplier tube during the thermal scans did show a sigmoidal behavior, suggesting the irreversibility of the transitions [23].

#### (d.3) Chemical denaturations

Chemical denaturations were performed at pH 7.0 and 25°C, by preparing samples at different urea concentrations, ranging from 0 to 6 M urea. The experiments were repeated twice. Reversibility experiments were carried out by dissolving the protein in 8 M urea, and diluting this stock solution until the corresponding denaturant concentration was obtained. Experiments were fully reversible.

#### (d.4.) Fitting of thermal and chemical denaturations

Although the thermal denaturations, either followed by fluorescence or CD were irreversible, we obtained the thermal denaturation midpoint, *T*_m_. The change in the physical property, *Y* (fluorescence intensity or the ellipticity), was fit to:
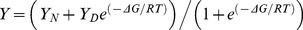
(2)where *Y*_N_  =  α_N_ + β_N_[T] and *Y*_D_  =  α_D_ + β_D_[T] are the baselines of the folded and unfolded states, respectively, for which a linear relationship with temperature is assumed; *R* is the gas constant; and *T* is the temperature in K. This equation was also used for the chemical-denaturations, where a linear relationship with denaturant concentration, [U], was assumed: *Y*_N_  =  α_N_ + β_N_[U] and *Y*_D_  =  α_D_ + β_D_[U].

The change in free energy, Δ*G*, for the thermal denaturations is given by:
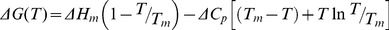
(3)where Δ*H*_m_ is the van't Hoff unfolding enthalpy; and Δ*C*_p_ is the heat capacity change of the folding reaction. The shape of Eq. 3 does not impose restrictions on the value of the Δ*C*_p_.

The chemical denaturation curves were analysed by using the two-state model for the unfolding reaction, according to: Δ*G*  =  *m*([urea]_50%_ – [urea]), where Δ*G* is the free energy of the unfolding reaction; [urea]_50%_ is the denaturant concentration at the midpoint of the transition; and *m* is the slope of the transition.

Fittings to Eqs. 2 and 3 were carried out by using the general curve-fit option of Kaleidagraph (Abelbeck software) running on a PC computer.

### (e) Dynamic light scattering (DLS)

DLS measurements were carried out in a NanoSizer ZS (Malvern Instruments, UK), operating a laser of 632.8 nm at an angle of 173°. The autocorrelation function was processed by using the instrument software in the CONTIN mode. This procedure provides a distribution of the hydrodynamic radii, *R*_h_, determined from the diffusion coefficient, *D*, using the Stokes-Einstein equation, 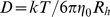. The solvent viscosity at 20°C was 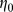 = 1.0031 cP. The experiment consisted of 12 measurements of 11 runs each one, with a length of 30 seconds *per* run, which amounted 11 minutes of data acquisition time. The CONTIN analysis allows for two modalities of analysis, one giving the fraction of intensity of the peaks, and the other the volume of those. The former is more sensitive and detects clearly the presence of large particles (even if their population is very small), while the latter detects the species that occupy a greater volume.

### (f) Analytical ultracentrifugation (AUC)

AUC experiments were performed in a Beckman Coulter Optima XL-I analytical ultracentrifuge (Beckman-Coulter, Palo Alto, CA, USA) equipped with UV-visible absorbance as well as interference optics detection systems, using an An50Ti 8-hole rotor, 12-mm-path-length charcoal-filled Epon double-sector centrepieces. The experiments were carried out at 20 °C, in the same solvent (90% H_2_O/10% D_2_O) as in NMR measurements. Laser delay was adjusted prior to the runs to obtain high-quality interference fringes. Light at 655 nm was used in the interference optics mode, and at 280 nm in the absorbance optics one. Sedimentation velocity runs were carried out at a rotor speed of 45000 rpm using 400-µl samples in the above mentioned solvent as reference.

A series of 400 scans, without time intervals between them, were acquired for each sample. A least squares boundary modeling of the sedimentation velocity data was used to calculate sedimentation coefficient distributions with the size-distribution c(s) method [24] implemented in the SEDFIT v13.0b software.

To express the results, the s_20,w_, solvent density (ρ = 1.0089 g/ml) and viscosity (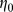 = 1.002 cP) at 20°C were estimated from the solvent composition using SEDNTERP software [25]. The partial specific volume of the protein, 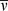, was 0.736 ml/g.

### (g) Size exclusion chromatography (SEC)

Analytical gel filtration experiments were performed in a Superdex 75 HR 10/30 column (GE Healthcare, Spain) at 1 ml/min and at 25°C on an AKTA FPLC system (GE Healthcare, Spain) following absorbance at 280 nm. A volume of 100 μl of HPr^bs^, at 11 and 22 μM, was loaded into the column after equilibration with 50 mM buffer Tris, pH 7.0 and 150 mM NaCl (to avoid protein-column interactions).

The column was calibrated by using the gel filtration low-molecular-weight calibration kit (GE Healthcare, Spain), as described [26]. The reported elution volumes for HPr^bs^ are the average of three measurements.

The elution of a macromolecule in gel filtration experiments is given by the partition coefficient, which is defined as the fraction of solvent volume within the gel matrix accessible to the macromolecule [27]. The average weight partition coefficient (σ) of protein standards and of HPr^bs^ were calculated by: 
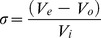
, where *V*_e_ is the elution volume of the corresponding protein, and, *V*_o_ and *V*_i_ are the void and internal volumes of the column, with values of 7.34±0.06 ml and 18.72±0.03 ml, respectively. The *V*_o_ and *V*_i_ volumes were determined, respectively, by using Blue dextran (5 mg/ml,) and riboflavin (0.5 mg/ml) by averaging three measurements for each compound.

The σs were determined for the molecular-size standards, and related to their molecular Stokes radius, *R*_s_, by using [27]:
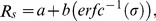
(4)where *a* and *b* are the calibration constants for the column, and *erfc* is the complementary Gauss error function used in probability and statistics.

Fitting of the calculated 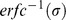 for the protein standards to Eq. 4 was carried out with Kaleidagraph (Abelbeck software) working on a PC computer. The *R*_s_ of any macromolecule can be determined, when *a* and *b* are obtained.

### (h) Isothermal Titration Calorimetry (ITC)

ITC titrations were carried out using an isothermal titration calorimeter Auto-ITC200 (MicroCal, GE Healthcare). To minimize the amount of gas present in the solution, the samples were degassed for 5 min at room temperature before being loaded into the calorimeter. Binding experiments were carried out titrating 15 μM HPr^bs^ within the sample cell (0.2007 ml) with wild-type EIN^sc^ (at 150 μM) and EINH186D [19] (at 200 μM) in the syringe. Binding reactions involving EIN^sc^-derived peptides [20] (and also with an equimolar mixture of both peptides) were performed; in this case, the sample cell was loaded with HPr^bs^ (at 20 μM) and the syringe was loaded with the corresponding peptide or their mixture (at 300 μM).

In all the assays, a total of 19 injections of 2 μl were added sequentially to the sample cell after a 150-s spacing to ensure that the thermal power returned to the baseline before the next injection. The amount of thermal power required to maintain the sample cell at a constant temperature after each injection was monitored as a function of time. The isotherms (the normalized heat upon binding *versus* the molar ratio of the reagents in the cell) were fit to a single-site model assuming that the complex has a 1∶1 stoichiometry. Data were analyzed with Origin 7.0 (OriginLab). As control experiments, the individual dilution heats for peptides and proteins were determined under the same experimental conditions by carrying out identical injections of the corresponding reagent into the sample cell, which contained only buffer. Dilution experiments with HPr^bs^ (at 200 μM) were carried out in a similar manner to test its self-association properties.

To determine the buffer-independent enthalpy of the binding reaction, the effect of the buffer ionization heat was taken into account by conducting the binding reaction in two buffers: (a) 10 mM Mes (pH 7.0); and, (b) 10 mM Mops (pH 7.0), which have different ionization enthalpies, Δ*H*_ion_ (3.55 and 5.05 kcal/mol, respectively). With this procedure [28], the buffer-independent enthalpy of the binding reaction, Δ*H* ^0^, and the number of exchanged protons between the complex and the bulk solution, *n*_H_, were calculated from linear regression of the observed enthalpy change.


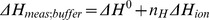
 (5).

The errors considered in the measured parameters (15% for *K*_D_ (or the self-association constant), 5% for Δ*G* ^0^ and 10% for Δ*G* ^0^, -*T*Δ*S* and *n*_H_) were taken as larger as the standard deviation between replicates and the numerical errors after fitting analysis.

### (i) Trypsin digestion

HPr^bs^ and lysozyme in 2 mM Tris pH 7.0 (200 μl containing 200 μg of protein) were incubated in the presence of trypsin (0.25 μg) at 37°C for 2 hours. Samples were retrieved at different intervals; the hydrolysis reaction was stopped by adding SDS-PAGE loading buffer and incubating the samples at 100°C for 5 min. Samples were loaded into an 18% gel to analyze trypsin digestion.

### (j) Cross-linking experiments

Purified HPr^bs^ aliquotes (200 μl) were incubated with 0.5% (vol/vol) glutaraldehyde (Sigma) in 50 mM Tris buffer (pH 7.0). The reaction mixture was incubated for 30 min at room temperature and reaction was stopped by boiling them during 5 min under standard denaturing conditions, for SDS-PAGE and samples were analyzed in 16% acrylamide gels. Gel images were acquired in a TDI GelPrinter Plus system connected to a PC computer.

### (k) NMR spectroscopy

The NMR data were acquired at 25°C on a Bruker Avance DRX-500 spectrometer (Bruker GmbH, Germany), equipped with a triple resonance probe and z-pulse field gradients. Processing of spectra was carried out with the XWINNMR software. Protein concentration was 30 μM.

#### (k.1) 1D-NMR experiments

Sodium trimethylsilyl-[2,2,3,3-^2^H_4_]-propionate was used as the internal chemical shift reference in the 1D-NMR spectra. Water was suppressed with the WATERGATE sequence [29]. Usually, 512 scans were acquired with a spectral width of 12 ppm, with 16 K data points in the time domain. The data matrix was zero filled to 32 K during processing.

#### (k.2.) Translational diffusion NMR experiments (DOSY)

Every DOSY measurement was repeated twice. Translational self-diffusion measurements were performed with the pulsed-gradient spin-echo sequence. The following relationship exists between the *D* and the acquisition delays [26]: 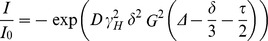, where *I* is the measured peak intensity of a particular (or a group of) resonance(s); *I*_0_ is the maximum peak intensity of the same resonance(s) at the smaller gradient strength; *D* is the translational self-diffusion constant (in cm^2^ s^−1^); δ is the duration (in s) of the gradient; *G* is the gradient strength (in T cm^−1^); Δ is the time (in s) between the gradients; γ_H_ is the gyromagnetic constant of the proton; and τ is the recovery delay between the bipolar gradients (100 μs). Data were plotted as *I*/*I*_0_ *versus G* ^2^, and the exponential factor of the resulting curve is 
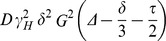
, from where *D* can be easily obtained. The duration of the gradient was 2.2 ms, and the time between both gradients was 150 ms. The methyl groups between 1 and 0.20 ppm were used for integration. The gradient strength was calibrated by using the value of *D* for the residual proton water line in a sample containing 100% D_2_O in a 5-mm tube [27]. The *R*_h_ of the protein was obtained by assuming that the *R*_h_ of dioxane is 2.12 Å [30], [31].

#### (k.3.) T_2_-measurements

Measurements of the *T*_2_ (transverse relaxation time) provide a convenient method to determine the molecular mass of a macromolecule, since the correlation time, τ_c_, is approximately equal to 1/(5*T*_2_) [32], with an inherent uncertainty of 10%. We measured the *T*_2_ for HPr^bs^ with the 1-1 echo sequence [33], and from that, we determined the τ_c_ and the molecular mass (which is, roughly, twice the correlation time, in aqueous solution, and for a typically hydrated and not-too-elongated protein) [33].

### (l) Modelling of the EIN^sc^:HPr^bs^ complex

The sequences of HPr^bs^ from (PTHP_LYSSH) and of EIN^sc^ from (PT1_STRCO) were extracted from the UniProtKB/Swiss-Prot server (www.uniprot.org/uniprot/), with extraction codes Q84F84 and Q9KZP1, respectively. The NMR structure of the EIN:HPr complex from *E. coli* [21] was used as structural template (PDB code 3EZA). Residues 82–89 of HPr^bs^ were modelled from PDB 3OQO (the complex of HPr of *B. subtilis* with the catabolite control protein), since the last residues of HPr (Leu-Ala-Gln) are missing in the 3EZA structure. The sequence alignments of EIN^sc^ and HPr^bs^ with those from *E. coli* are displayed in Fig. S1 in File S1. The pairwise alignment for EIN^sc^ was extracted from a multiple alignment of EIN from several species performed by using BLAST (http://blast.ncbi.nlm.nih.gov). In both for EIN^sc^ and HPr^bs^, the sequence similarity with other EIN and HPr species is ∼60%, while there is also structural conservation among species. This is the best possible scenario to develop reliable *in silico* homology models [34], [35].

The molecular system was described in terms of internal coordinates, as implemented in the ICM program [36] (www.molsoft.com). Force-field parameters and vacuum energy terms were taken from the ECEPP/3 force field [37]. The solvation-energy contribution was calculated as implemented in ICM, where the non-polar contribution to the solvation energy was assumed to be proportional to the solvent-accessible surface area (surface-tension parameter, γ = 12 kcal mol^−1^Å^−2^). The strain stemming from non-identical amino acid substitution in the EIN^sc^:HPr^bs^ complex was relieved through restraint local energy minimization [38], to prevent significant structural distortion.

### (m) Optimization of the binding interface through protein-protein docking

Considering EIN^sc^ as the receptor and HPr^bs^ as the ligand, their interaction was optimized by docking the ligand onto the receptor, using a full flexible Monte Carlo-based stochastic global energy minimization [39], [40]. The six rigid coordinates of the ligand and the torsion angles of the residues at the interface were considered free during the simulations. The corresponding residues of EIN^sc^ at the interface were: Gly66, Glu67, Ala68, Gln69, Ala70, Val71, Glu73, Ala74, Met77, Met78, Ala79, Asp81, Glu83, Leu84, Asp87, Val88, Val102, Phe106, Tyr109, Arg110, Leu113, Tyr119, Leu120, Arg123, Val124, Leu127, Asp128 and Arg131. The HPr^bs^ residues at the interface were (using prime notation for HPr^bs^): Val8′, Asp10′, Leu12′, Gly13′, Ile14′, His15′, Ala16′, Arg17′, Pro18′, Ala19′, Ser20′, Leu22′, Val23′, Ala24′, Thr27′, Thr37′, Asn43′, Leu44′, Lys45′, Ser46′, Ile47′, Leu48′, Gly49′, Val50′, Met51′, Gly52′, Leu53′, Ala54′, Leu55′ and Phe61′.

The simulation temperature of the Monte Carlo-based protein-protein docking was set to *T* = 600 K to improve conformational sampling. An initial ensemble of 15 EIN^sc^:HPr^bs^ complexes was generated by randomizing the six torsion angles of the ligand. Each complex within the ensemble was subjected to a cycle of short local energy minimization steps using a soft van der Waals interaction energy term in which the strength of that interaction was gradually increased [40], [41]. The torsion angles of the residues at the interface and the six positional coordinates of HPr^bs^ were set free during the minimization. A stochastic global energy optimization was then performed for each of the 15 complexes. A total of 10 million energy evaluations were allowed and low-energy conformations were stored in a conformational stack [42]. Redundant conformations with respect to the backbone atoms of the ligand molecule were eliminated by using a low threshold of 0.7 Å. Due to the inaccuracies in the force-field approximation and the lack of an *ad hoc* parameter optimization, the low-energy conformational states collected during the simulation were inspected, although all show the same conformational trend.

## Results

### The HPr^bs^ is a well-folded protein at physiological pH

**Figure 1:**
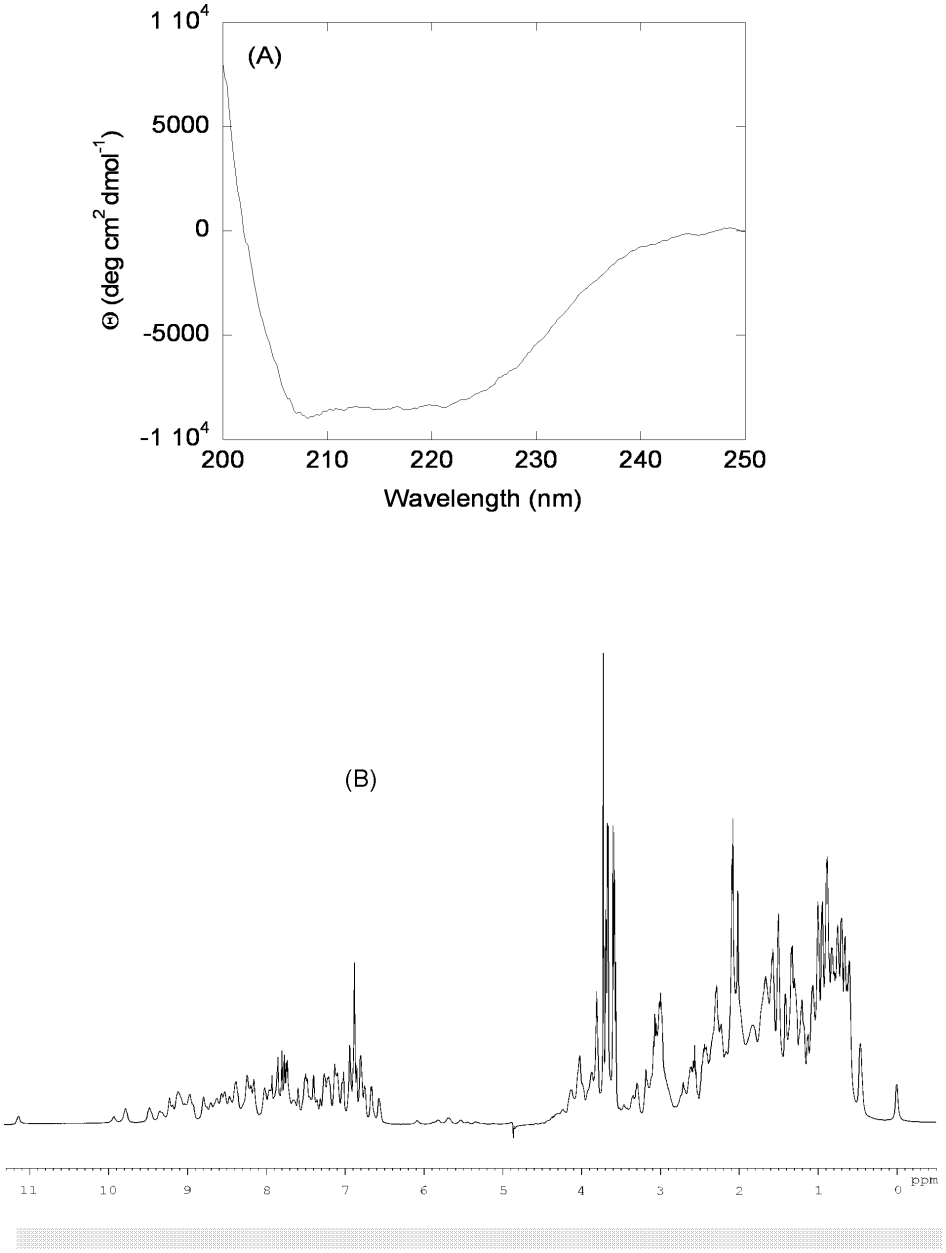
Spectroscopic features of HPr^bs^ at physiological pH. (A) Far-UV CD spectrum of HPr^bs^ at physiological pH; protein concentration was 15 μM. (B) 1D-^1^H-NMR spectrum of HPr^bs^ in the amide (left side) and methyl (right side) regions. Protein concentration was 50 μM. Experiments were carried at 25°C.

The fluorescence spectrum of HPr^bs^ had a maximum at 305 nm, corresponding at the emission of the sole tyrosine residue (data not shown). The near-UV CD spectrum of the protein was null, due to the low percentage of aromatic residues of the protein (HPr^bs^ has only a tyrosine and four phenylalanines). The far-UV CD spectrum of HPr^bs^ is similar to those of other HPr species [19] (Fig. 1 A). Deconvolution of the far-UV CD by using K2d (http://www.embl.de/~andrade/k2d/) yielded a 42% of α-helix, a 27% of β-sheet and a 31% of random-coil. Furthermore, the modelled structure of HPr^bs^ (see below) yielded a 34% of α-helix, a 30% of β-sheet, a 24% of random-coil, and a 4% of turns. Thus, we conclude that the structure of HPr^bs^ is similar to that of other members of the family.

The 1D ^1^H-NMR spectrum of HPr^bs^ shows the presence of: (i) upfield-shifted methyl groups; and, (ii) a large spreading of the signals in the amide region (Fig. 1 B). Furthermore, there are some down-field shifted H_α_ protons between 6.1 and 5.4 ppm (Fig. 1 B), which indicate the presence of β-sheets. All these features correspond to the spectrum of a well-folded protein [43] with β-sheet structure.

We also carried out trypsin digestion experiments (Fig. S2 in File S1), and we observed that, when compared to lysozyme, HPr^bs^ was more easily digested. These results suggest that: (i) HPr^bs^ was not as stable as lysozyme; or alternatively (ii) the trypsin cleavage sites were more solvent-exposed than in lysozyme (see Discussion).

To sum up, we conclude, from the spectroscopic probes, that the HPr^bs^ at pH 7.0 is folded.

### The HPr^bs^ self-associates

Since HPr^bs^ showed a tendency to aggregate during purification, we tested whether the protein was oligomeric at pH 7.0 by using different techniques.

**Figure 2:**
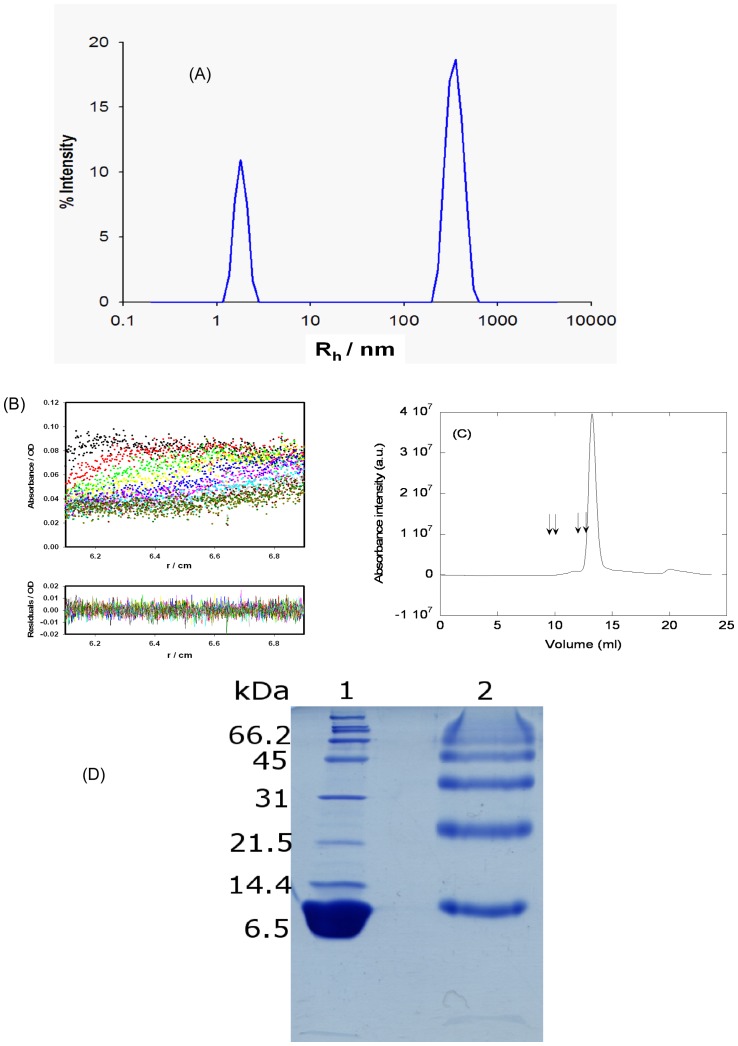
Hydrodynamic features of HPr^bs^. (A) The CONTIN analysis of the DLS experiment where the protein size distribution is shown as intensity. (B) Example of the AUC absorbance scans at 11 times of centrifugation, from 3447 s (top, in black) to 60556 s (bottom, in green), with the same time interval between scans. It is important to note the initial, baseline absorbance of only 0.08 units. On the second panel, the residuals of SEDFIT fitting for each time are shown; it is important to note that the residuals are large but random, without any anomalous systematic trend. (C) SEC profile, as monitored by the absorbance at 280 nm, of the main peak of HPr^bs^ in a Superdex 75 HR 10/30. Other peaks were observed at larger elution volumes (larger than 19 ml, the bed volume of the column, as it can be observed on the right side of the panel) probably due to protein-column interactions. The arrows indicate the position of the protein standards used in column calibration, from left to right: bovine serum albumin (9.31 ml), ovoalbumin (10.29 ml), chymotrypsinogen (12.33 ml) and ribonuclease A (13.14 ml). (D) Cross-linking experiments: Left lane: The BioRad marker with the indicated molecular weights. Right lane: the cross-linked HPr^bs^.



First, we used DLS (Fig. 2 A). The distribution by intensity shows a peak in the range 200 – 500 nm, which demonstrates the mentioned tendency of the particle to self-associate (see Materials and Methods section), but it also displays a peak at 1.77±0.06 nm, which clearly corresponds to the monomeric protein. However, this latter peak showed a large polydispersity (34%), suggesting the presence of equilibria involving the monomeric protein species. From the molecular weight of the protein, 10921.5 Da as calculated from the amino acid sequence, one could make a guess of the expected *R*_h_ from the classical theory of hydrodynamics of elongated (ellipsoidal) hydrated particles.

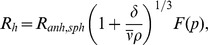
where δ is the degree of hydration (usually 0.3 g(water)/g(protein)), *R_anh,sph_* is the radius the particle would have if it was spherical and anhydrous, 
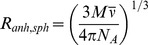
, and *F*(*p*) is a correction factor. Taking into account the values of 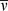 and ρ described, we obtained for a hydrated sphere (p = 1, F(p) = 1) a R_h_ of 1.65 nm. Some deviation from the spherical shape (about p = 2) would match this value with the experimental one.

The peak from DLS experiments has an appreciable width (Fig. 2 A), but this must be due to: (i) the well-known tendency of the CONTIN analysis to give artificial wide peaks; or (ii) sample polydispersity (34%), which could be the result of the presence of several species in equilibrium.

Second, we carried out AUC measurements. The AUC experiment was affected by the low concentration of the species in solution. After filtering, the remaining aggregates of several hundred nanometers sediment immediately and, therefore, they were not detected; the sample concentration present in solution was 70 μM. Since HPr^bs^ has a single Tyr residue, the AUC signal was very low; the initial absorbance at 280 nm was 0.08 units only, when the lower limit for a proper measurement is usually considered 0.1 units. In fact, the AUC sedimentation profiles were noisy (Fig. 2 B). Yet the experiment was feasible, and the outcome was analysed by using SEDFIT, yielding a broad band in the c(s) distribution, with s_20,w_ between 0.8 and 2.0 S. The excessive width of the distribution is attributed to the low signal-to-noise ratio of the data. The residuals of the fit (Fig. 2 B) are randomly distributed suggesting that the experimental data was not skewed by any artefact, but rather by the low signal-to-noise ratio. We also carried out measurements in the interference mode (data not shown), with the results reproducing very closely the spread of the sedimentation coefficient observed in AUC. Therefore, according to the fundamental equation of sedimentation velocity, 
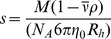
, by using the *R*_h_ from DLS for the monomeric species (see above), we obtain s_20,w_  = 1.5 S, which is within the range of observed values for monomeric species. Unfortunately, due to the low signal-to-noise ratio of the AUC data, we could not rule out the presence of oligomers, whose possible s_20,w_ values would not be much different from that of the monomer (for instance, the s_20,w_ ratio of a dimer of spherical units to the monomeric unit is close to 1.5).

In the SEC experiments, the samples eluted as a single major peak at 13.37 (at 11 μM) and 13.24 ml (at 22 μM), respectively, but also other peaks appeared at larger volumes than the bed volume of the column (19 ml) (Fig. 2 C). It could be thought that those differences in the elution volume at the two concentrations explored are small, but we have observed similar differences in other self-associating proteins when using slightly different protein concentrations in high resolution SEC [26]. These results suggest that: (i) there are protein-column interactions; and, (ii) that the elution volume is concentration-dependent in the range of protein concentrations explored. Calculation of the corresponding *R*_s_ yields 1.60 and 1.65 nm, respectively. These values are very similar to those experimentally obtained from DLS for the monomeric species.

The DOSY measurements yielded a *D* of (1.06±0.02) 10^−6^ cm^2^ s^−1^ for HPr^bs^, which resulted in a *R*_h_ of 1.62±0.03 nm (for a *D* of dioxane of (8.08±0.04) 10^−6^ cm^2^ s^−1^). This value is similar to that obtained by DLS; however, it is important to indicate that DOSY-NMR reports on the average populations in solution, and that large molecular weight species (as those detected by DLS) are not observed in the NMR spectrum due to signal broadening (this lack of detection of large molecular weight proteins in the NMR spectrum would also explain the well-spread and sharp 1D-1H-NMR spectrum, Fig. 1 B).

The *T*_2_-relaxation measurements indicate that the signals, corresponding to amide protons of HPr^bs^ appearing at 10.20 and 9.8 ppm have a *T*_2_ of 29 and 27 ms, which yield τ_c_s of 6.8 and 7.4 ns, respectively. These values lead to estimated molecular weights of 13.6 and 14.8 kDa. Given the error of the technique (10%), we can say that those amide protons correspond to a monomeric protein.

We carried out cross-linking experiments with glutaraldehyde. The 16% SDS-PAGE showed bands at 9 kDa (which would correspond to the monomeric species), 22 kDa, 37 kDa and 53 kDa (Fig. 2 D). The largest species detected in these experiments would probably correspond to the first peak detected by DLS experiments, which has a large polydispersity (34%), suggesting the presence of different species in equilibrium with the monomeric one (Fig. 2 A).

Finally, we also used the ITC technique. Dilution experiments showed the presence of a self-association equilibrium (Table 1). Attempts to fit this isotherm to an indefinite self-associating species [44] (given the size of the species detected by DLS and the cross-linking experiments) failed, and therefore, we fitted the isotherm to the most simple possible equilibrium which could be prevalent among other reactions (dimer↔monomer). The apparent self-dissociation constant was 6.5 µM and the process is entropically driven as given by the values of enthalpy and entropy (Table 1).

**Table 1:** Thermodynamic parameters for the binding reactions as determined by ITC^a^_._

Complex	*K*_D_ (µM)^b^	Δ*H* ^0^ (kcal/mol)^d^	Δ*G* ^0^ (kcal/K·mol)^e^	−TΔ*S* (kcal/mol)^f^	*n*_H_^g^
HPr^bs^	6.5^c^	−5.7	−7.05	−1.3	−0.97
HPr^bs^: wild-type EIN^sc^	1.9	−0.2	−7.80	−7.6	0.17
HPr^bs^:EINH186D	2.9	1.6	−7.50	−9.1	−0.31
HPr^bs^: EINbsite	3.9	−1.4	−7.40	−6.0	−0.31
HPr^bs^: EINosite	3.0	−0.5	−7.50	−7.0	0.28
HPr^bs^: (EINbsite + EINosite)	4.8	0.3	−7.30	−7.6	−0.02

a All titrations were conducted in Mes and Mops buffers 10 mM (pH 7.0) at 25°C. ^b^ *K*_D_ is the dissociation constant; ^c^ *K*_D_, for HPr^bs^ dilution, is the self-dissociation constant. Typical relative errors are 15% for *K*_D_, 5% for Δ*G* ^0^ and 10% for Δ*H* ^0^, −TΔ*S* and *n*_H_. Experiments were performed in duplicate. ^d^Δ*H* ^0^ is the buffer independent enthalpy for the binding reaction; its value was determined by conducting experiments in Mes and Mops buffers, 10 mM (pH 7.0) at 25°C, and by using Eq 5. ^e^Δ*G* ^0^ is the binding free energy at 25°C, determined as Δ*G* ^0^  =  RT ln *K*_D_. ^f^−TΔ*S* ^0^ is the value of the entropic contribution of the binding reaction at 25°C, determined as −*T*Δ*S* ^0^  =  Δ*G* ^0^ – Δ*H* ^0^. ^g^ *n*_H_ is the number of exchanged protons, determined by conducting experiments in Mes and Mops buffers, 10 mM (pH 7.0) at 25°C, by using the Eq. 5.

Taken together, most of the hydrodynamic techniques show the presence of a monomeric species, which are the most populated under the conditions explored. However, there is evidence from DLS, SEC, ITC and cross-linking experiments of self-associated species.

### The HPr^bs^ maintains its secondary structure in a wide pH interval

**Figure 3:**
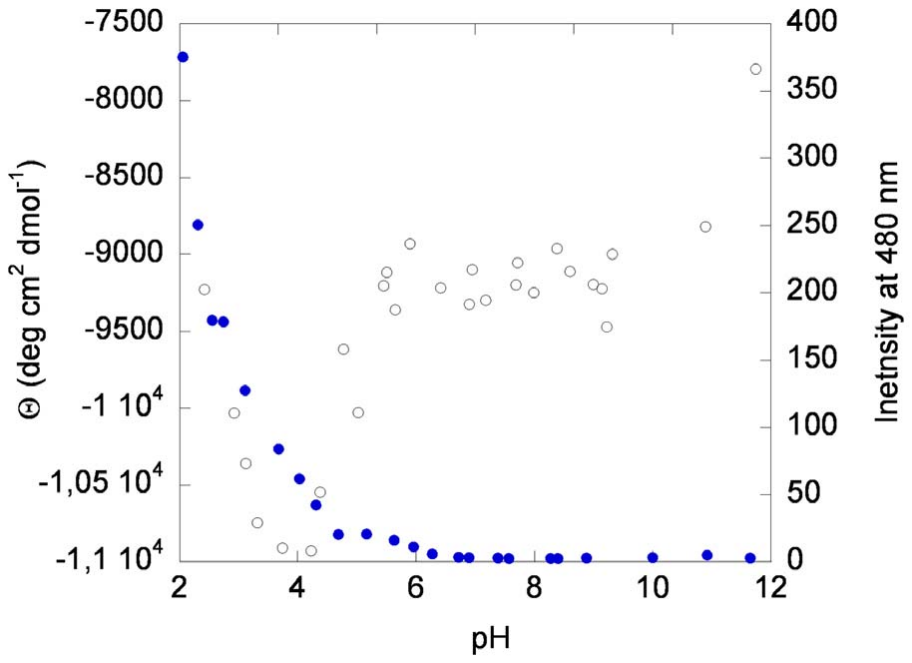
The pH-denaturation of HPr^bs^. Changes in ellipticity at 222 nm (left side, blank circles) and in ANS-binding at 480 nm (right side, blue filled circles) upon pH variation are shown. Experiments were carried out at 25°C with protein concentration of 15 μM in both techniques.

Next, we carried out the study of the structural changes with pH, to see whether the behaviour is similar to that reported in HPr^sc^ [15]. We did not use intrinsic fluorescence to monitor the pH-structural changes, since the tyrosine only reports its own titration occurring at basic pHs (data not shown). The pH-denaturation, followed by the changes in ellipticity in the far-UV CD at 222 nm, shows two transitions (Fig. 3). One at pH lower than 4, and for which we could not determine its p*K*_a_ due to the absence of acidic baseline; and the other with a p*K*_a_  = 5.2±0.3. Interestingly enough, this latter titration resulted in a decrease (in absolute value) of the ellipticity at 222 nm (and thus, in a decrease of helical structure [45], [46]). The secondary structure of the protein did not change from pH 7.0 to 10.0.

ANS-binding is used to monitor the extent of solvent-exposure of protein hydrophobic regions, and to detect the existence of non-native partially folded conformations. When ANS is bound to solvent-exposed hydrophobic patches of proteins, its quantum yield is enhanced and the maxima of the emission spectra are shifted from 520 nm to 480 nm [47]. At low pH values, the intensity of the ANS fluorescence spectrum in the presence of HPr^bs^ was largely enhanced and the maximum wavelength appeared at 482 nm (Fig. 3). As the pH was increased, the spectral intensity was reduced and the maximum wavelength shifted towards 528 nm. These results suggest that: (a) ANS was bound to HPr^bs^ at low pH values, probably because of the presence of solvent-exposed hydrophobic regions; and, (b) those hydrophobic patches were buried in the pH range 2.0–5.0, as concluded from the titration observed; we could not determine the p*K*_a_ of this titration due to the absence of acidic baseline. Since the species at low pH had a larger ellipticity than the native one at pH 7.0, and it was bound to ANS, we suggest that it is a molten-globule [48]. A similar behaviour has been observed in HPr^sc^ [15].

### The HPr^bs^ folds irreversibly by heat

Given that the protein acquires a well-folded structure at pH 7.0, we carried out the study of the conformational stability by thermal and chemical denaturants at this pH.

#### (a) Thermal denaturations

**Figure 4:**
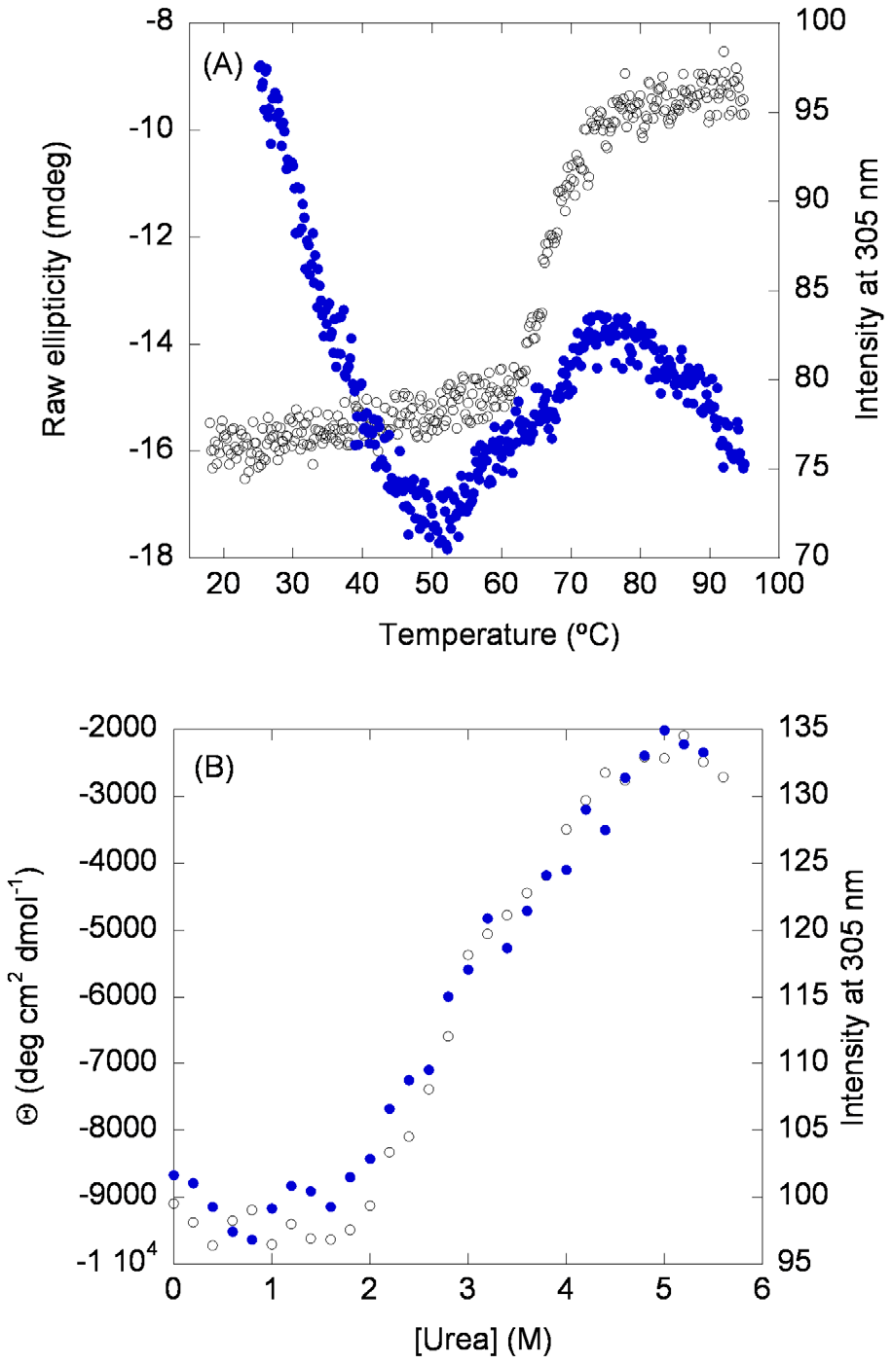
Thermal and chemical denaturations of HPr^bs^. (A) Thermal denaturations followed by the changes in ellipticity at 222 nm (left side, blank circles) and in the intrinsic fluorescence emission at 305 nm, after excitation at 278 nm (right side, filled blue circles). (B) Urea-denaturations followed by the changes in ellipticity at 222 nm (left side, blank circles) and in the intrinsic fluorescence emission at 305 nm (similar results were obtained at 315 and 330 nm), after excitation at 278 nm (right side, blue filled circles).

Thermal denaturations, followed by the changes in ellipticity at 222 nm, yielded an irreversible transition with an apparent *T*_m_ of 66.7±0.4°C (Fig. 4 A, blank circles, left side). We cannot elaborate more on that value since the process was irreversible; however, it is interesting to note that the ellipticity at the highest temperature was not null, suggesting the presence of residual structure in the protein, as it occurs in other HPrs [15, 16 and references therein]. No thermal denaturations were observed at pH <5.0 and pH >9.0 (data not shown).

Thermal denaturations were also followed by fluorescence (Fig. 4 A, filled circles, right side). The long slopes in the native and unfolded baselines precluded the determination of an accurate *T*_m_ which was close to 67±3°C. Thermal denaturations did not show any protein-concentration-dependence (see Discussion).

#### (b) Chemical denaturations

The reversible urea-denaturations of HPr^bs^ were followed by far-UV CD and fluorescence (Fig. 4 B). The changes in ellipticity at 222 nm followed a sigmoidal behaviour (Fig. 4 B, blank circles, left side), with a [urea]_50%_  = 2.7±0.1 M, and very low *m*-value (1.0±0.2 kcal mol^−1^ M^−1^), which pinpoints to a low cooperativity of the transition. The fluorescence data (Fig. 4 B, filled circles, right side), obtained by following the intensity at 305 nm, showed a larger scattering, with a [urea]_50%_  = 2.8±0.5 M. The fact that both techniques (which provide dissimilar information on the structure) yielded the same [urea]_50%_ suggests that the chemical denaturation of HPr^bs^ is a two-state process. As with thermal denaturations, chemical ones were not protein-concentration-dependent.

### Affinity of HPr^bs^ for wild-type EIN^sc^, phosphorylated EIN^sc^ and EINsite peptides

**Figure 5:**
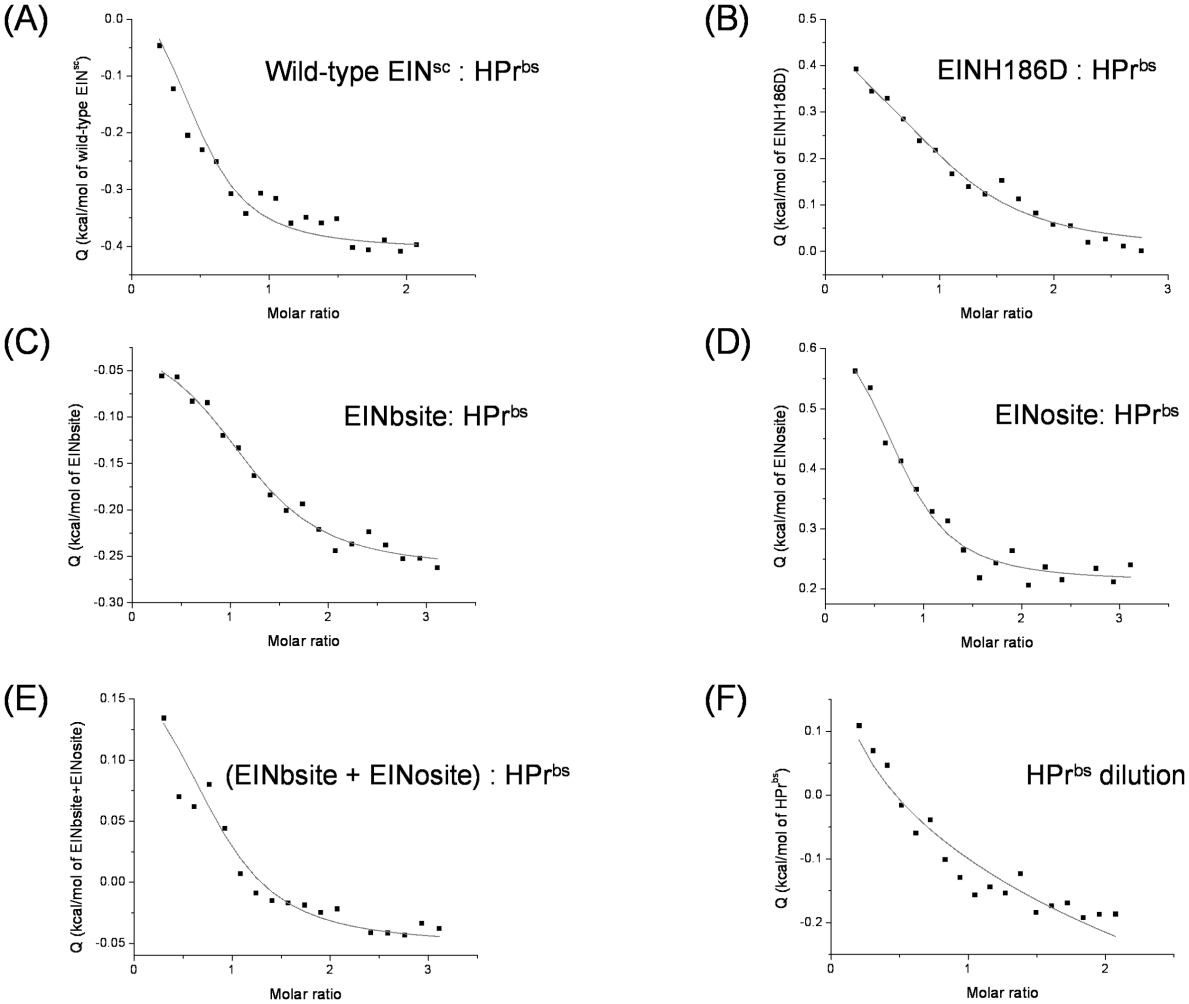
Determination of the affinity constants of the complexes by ITC. The fitting curves were obtained assuming a 1∶1 model. Binding curve for the titration between: (A) wild-type EIN^sc^:HPr^bs^; (B) EINH186D:HPr^bs^; (C) EINbsite:HPr^bs^; (D) EINosite:HPr^bs^; (E) (EINbsite+EINosite):HPr^bs^ (ligands were at equimolar concentrations); and, (F) HPr^bs^ dilution. All the titrations were carried out at 25°C in Mes buffer, 10 mM (pH 7.0).

The affinities of HPr^bs^ for EIN^sc^ (either wild-type or EINH186D), and for EINsite peptides were determined by using ITC (Table 1, Fig. 5). Since HPr^bs^ showed dilution effects (Fig. 5 A), in all the binding experiments, HPr^bs^ was loaded into the sample cell to minimize the effect of its dilution during the binding reaction. The *K*_D_s of the complexes involving wild-type EIN^sc^ or EINH186D were similar (Table 1); this result is surprising, since one should expect a lower affinity for the EINH186D. However, similar *K*_D_s were also observed in the interaction of (wild-type or mutant) EIN^sc^ with HPr^sc^ [19]. The *K*_D_s of the complexes of HPr^bs^ with the EINsite peptides (either isolated or as a mixture) were two-fold higher than that of the intact proteins. We used mixtures of peptides to see whether there was a synergy between both binding sites. Since the *K*_D_ for the mixture was similar to that of the separated peptides, we conclude that there is no synergy between both peptides.

The values of the enthalpies and entropies indicate that all the binding reactions were entropically driven (Table 1); this result indicates a main favourable contribution from hydrophobic desolvation in the binding reaction. On the other hand, a positive value of *n*_H_ indicates a protonation (that is, the uptake of a proton by the complex) as occurred in the EIN^sc^:HPr^bs^ and in the EINosite: HPr^bs^ complexes (Table 1). A negative value of *n*_H_ indicates a deprotonation (that is, the release of a proton from the complex), as it occurred in the complexes with EINH186D and EINbsite. In the formation of the complex of HPr^bs^ with a mixture of both EINsite peptides, there was no proton exchange between the complex and the bulk solution.

### Computational structural characterization of the EIN^sc^:HPr^bs^ complex

Since we have shown experimentally that there was binding between HPr^bs^ and the wild-type EIN^sc^, we decided to model the structure of the complex. Furthermore, the structure of the modelled HPr^bs^ could give insights into the structural location of the trypsin cleavage sites.

**Figure 6:**
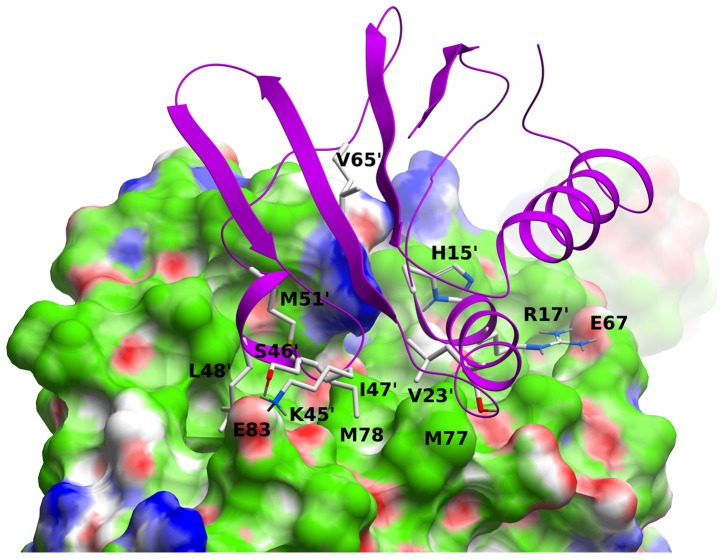
Molecular modelling of the EIN^sc^:HPr^bs^ complex. The EIN^sc^ is shown in skin representation, color code: green, hydrophobic; red, hydrogen bond acceptor; blue, hydrogen bond donor. The HPr^bs^ protein is shown as a magenta ribbon, and key residues are explicitly displayed. Numbers without the prime symbol correspond to EIN^sc^ numbering, and numbers with the prime symbol are those from HPr^bs^. The figure was obtained with ICM [36].

The best representative modelled structure of the complex is shown on Fig. 6, along with a comparison of the sequences used in the modelling. The interface residues are mainly conserved from the *E. coli* structure (PDB number 3EZA), with some exceptions that make the interface more hydrophobic. To describe the EIN:HPr interactions we use the following nomenclature: numbers without the prime symbol correspond to EIN^sc^, numbers in brackets correspond to EIN^ec^, and numbers with the prime symbol correspond to the HPr^bs^ numbering, which coincides with the HPr^ec^; arrows indicate EIN^ec^ to EIN^sc^, or HPr^ec^ to HPr^bs^ mutations. The conserved salt-bridges involve the following residues: Glu67 and Arg17′ (although the one involving (Glu67)->Gly66 and Arg17′ was lost), and Glu83 and Lys45′ (although the one between Glu83 (Glu84) and Lys49′->Gly49′ were lost). The following salt-bridges were also lost: Glu73 (Glu74) and Lys24′->Ala24′, and Asp81 (Asp82) and Lys27′->Thr27′, however, the hydroxyl group of Thr27′ establishes a hydrogen bond with the carbonyl oxygen of Val23′. As in the *E. coli* structure, the hydroxyl group of Tyr119 (Tyr122) and the guanidine group of Arg123 (Arg126) were within hydrogen bond distance of the carbonyl oxygen of Ile14′ (Leu14′)'. The hydrogen bond between Glu83 and Ser46′ was conserved, though the one between Asp126 (Asp129) and Thr16′->Ala16′ was lost. The lost electrostatic interactions, however, involved mainly exposed residues. Most hydrophobic residues at the interface were either conserved or have a conservative substitution: the strictly conserved residues were Ala70, Val71, Met77, Leu84, Leu113, Gly13′, Pro18′, Ala19′, Val23′. The conservative substitutions maintain or increase the hydrophobic contact at the interface: Gly(75)->Ala74, Leu(79)->Met78, Gln(111)->Tyr109, Ala(127)->Val124, Val(130)->Leu127, Asn12′->Leu12′, Leu14′->Ile14′, Thr16′->Ala16′, Leu47′->Ile47′, Phe48′->Leu48′, Gln51′->Met51′, Thr52′->Gly52′, and Gly54′->Ala54′. The hydroxyl group of Tyr109 (in EIN^sc^) is hydrogen-bonded to Asp87. In our model, EIN^sc^ and HPr^bs^ were phosphorylated at the active-site histidines, His186 (His189) and His15′. The His186 of EIN^sc^ did not show direct contacts with HPr^bs^ (as in EIN^ec^:HPr^ec^), and kept a conserved interaction with Thr165 (Thr168). This interaction could explain why no change in EIN^sc^:HPr^sc^ affinity was observed upon His186Asp mutation in EIN^sc^.

## Discussion

### The HPr^bs^ has a tendency to self-associate

One of the main conclusions of this work is the identification, for the first time, of an intrinsic tendency to self-associate in a wild-type HPr species under physiological conditions. The use of several biophysical techniques shows that wild-type monomeric HPr^bs^ exists in solution in equilibrium with other self-associated species. From the different used techniques, only ITC, SEC, DLS and cross-linking experiments showed the presence of self-associated species. The ITC showed the presence of self-associated species due to the heat associated with dilution of HPr^bs^; the Fig. 5 F shows the calorimetric dilution assay for HPr^bs^ (that is, HPr^bs^ at high concentration injected into buffer) to test whether or not HPr^bs^ associates in the native state. Because of the non-constant profile obtained in the thermogram, it is possible to conclude that native HPr^bs^ is not monomeric. From the analysis of those curves, an apparent dissociation constant can be estimated. The cross-linking experiments (Fig. 2 D) showed the presence of several high-molecular weight species together with the monomeric ones, as in the DLS results, where monomeric and species with a large *R*_h_ were detected (Fig. 2 A). Finally, the high resolution SEC experiments showed a concentration-dependence elution volume. In addition, we observed during the purification protocol, a high tendency of the protein to aggregate. We could not detect the presence of high-molecular weight species by NMR due to signal broadening;moreover, AUC could not distinguish between monomeric and other species (see above) due to the low signal-to-noise ratio. We do not know, however, if the self-associated species are specific or non-specific aggregates. However, since different batches of purified protein did always yield the same heat dilution during the ITC experiments, and the same SEC chromatograms were always obtained a specific self-associated species seem to be present.

A mutant of *B. stearothermophilus* HPr (at the Phe29 position, in the in-between loop of the first α-helix and the second β-strand) showed the presence of domain-swapped dimers in X-ray structures; this dimer, however, was not detected in solution by any hydrodynamic technique [49] (probably, because the concentrations used in the hydrodynamic measurements were not as high as those used during crystallization). The structure of the swapped-domain involves swapping of residues 56–88, the C-terminal β-strand and α-helix of one monomer, for the corresponding elements in the other monomer. This structure is very different to that observed for a homologous protein, Crh (catabolite repression HPr), from *B. subtilis*, where the swapped dimer is formed by the N-terminal first β-strand of each monomer [50]; however, the Crh shows the presence of a mixture of monomers and dimers even in solution [51]. These results suggest that, although they had not been detected so far in the wild-type species, HPr-like species can form dimers in solution. In HPr^bs^, however, there are also other self-associated species, apart from dimeric ones (Fig. 2 D).

**Figure 7:**
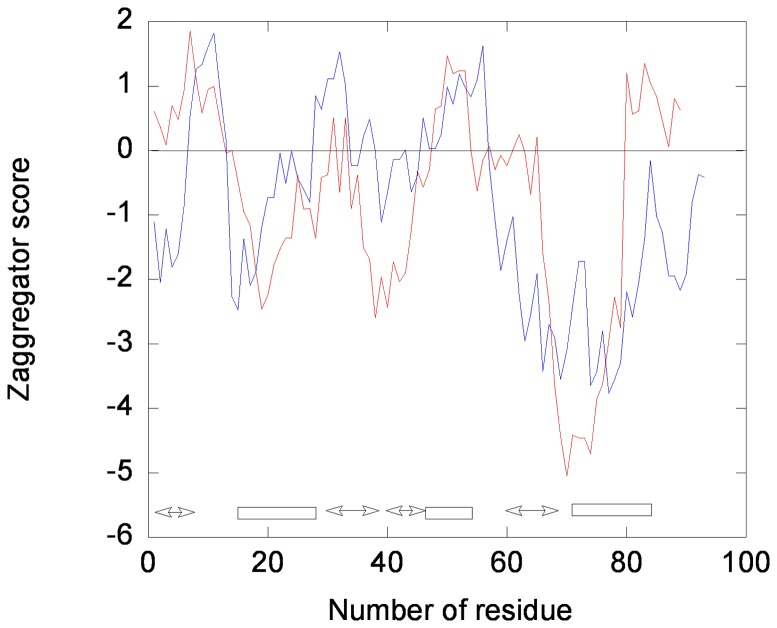
Aggregation propensity of two HPr species as predicted by Zyggregator. The plot shows the intrinsic aggregation propensity of HPr^bs^ (red, blank circles) and HPr^sc^ (blue, filled circles). Because of the way the scores are normalized, aggregation-prone regions have a score larger than one. A positive value of the score indicates an aggregation-prone region (especially when the value is larger than 1). The rectangles at the bottom indicate the α-helical regions, and the double-headed arrows the presence of β-strands.

We tried to rationalize these unexpected results by studying the propensities to aggregate of HPr^bs^ from the unfolded state with the Zyggregator algorithm [52], [53]. This method is based on a combination of the physicochemical properties of amino acids, including electrostatic charge, hydrophobicity and alternating hydrophobic-hydrophilic patterns. The algorithm leads to a pattern of positive or negative peaks depending of the protein sequence. Positive peaks (larger than one) in the intrinsic aggregation propensity profiles correspond to aggregation-prone regions, and negative peaks to aggregation-resistant regions. The Zyggregator profile of HPr^bs^ (Fig. 7) shows the presence of aggregation-prone regions, in the modelled structure, at the: (i) C-terminal α-helical region; (ii) N-terminal first β-strand; and, (iii) forties α-helical region. We also calculated the Zyggregator plot for the HPr^sc^, which is a monomer, and whose conformational stability and structural features have been described [15], [16]. There were also aggregation-prone regions in HPr^sc^, but only in the in-between loops of secondary structure elements. We also used other algorithms, such as Aggrescan [54], yielding similar results to those described here (data not shown).

The Zyggregator results suggest that there are two possible interacting regions in HPr^bs^: the N and C-termini. Interestingly enough, these were the two regions shown to form the different domain-swapped domains in *B. stearothermophilus* HPr and Chr [49], [50]. However, it is important to indicate that, at this stage, we do not have any evidence of domain-swapping in the high-molecular-weight species of HPr^bs^. The tendency of HPr^bs^ to self-associate through two regions (and not only to form dimers) might explain the DLS (Fig. 2 A) and cross-linking results (Fig. 2 D), where high-order molecular species were observed.

### The HPr^bs^ shows irreversible thermal denaturation

Other HPr members show reversible chemical- and thermal-denaturations, under all the conditions explored (see [15], [16], and [55], [56] and references therein). However, HPr^bs^ showed irreversible thermal denaturations. Although the irreversibility of the process precludes any further interpretation, we can speculate on the values of the *T*_m_. The apparent *T*_m_ at pH 7.0 was 66.7±0.4°C, which is similar to that observed in other HPr members [15], [16], [55], [56], suggesting that we are following (either by fluorescence or CD) the melting of the secondary and tertiary structure of the monomeric species.

There are several lines of evidence which could explain why we did not observe a protein concentration-dependence in the *T*_m_. First, the unfolded state of the protein has also a tendency to self-associate. Second, the range of protein concentrations explored is very small (although we observed a concentration-dependence in the elution volume in the SEC experiments in that range of explored concentrations, see above). And finally, dissociation is spectroscopically silent by fluorescence or CD, since it does not involves disruption of secondary structure (that is, the structure of the monomer is the same in isolation or becoming part of the high-molecular species) or modification of the environment around the sole tyrosine. Absence of protein concentration-dependence during thermal or chemical denaturations has been observed in other oligomeric proteins [57].

In the chemical denaturation experiments, the [urea]_50%_ (2.7±0.1 M) was similar to that of HPr^sc^ at physiological conditions (2.8±0.2 M) [15], [16]. Furthermore, the *m*-value is within the same range to that observed in other HPr species [16], [18], [55], [56] (1.0±0.2 kcal mol^−1^ M^−1^). In addition, this value was similar to that theoretically calculated from the accessible surface area of the modelled structure [58] (1.57 kcal mol^−1^ M^−1^), and therefore, it underpins that the secondary and tertiary structures of HPr^bs^ are similar to that of other HPrs. The small value of *m* (and thus a poor co-operative transition), together with the above suggestions for thermal-denaturation experiments, could also explain the absence of protein-concentration-dependence during the chemical denaturations. The figures of *m* and and [urea]_50%_ allow to estimate a free energy of 2.7±0.2 kcal mol^−1^, which is rather low even for a HPr member [16], [18], [55]. Since close inspection of the trypsin digestion sites in the modelled structure of HPr^bs^ and that of lysozyme shows that those sites are, in both proteins, solvent-accessible, this low stability of HPr^bs^ also explains the results of trypsin digestion, since lysozyme has a larger free energy (12 kcal mol^−1^ [59], [60]) than HPr^bs^ (3 kcal mol^−1^). We do not know yet whether this low stability of the isolated monomeric species triggers self-association.

On the other hand, HPr^bs^ shows a similar behaviour upon pH-denaturation as HPr^sc^ [16]; even the midpoint (p*K*_a_) of the transition monitored by far-UV CD in both proteins in the same, and close to the p*K*_a_ of solvent-exposed glutamic residues [61]. These results pinpoint that the structure of the monomer of HPr^bs^ is basically the same as that of other members of the family.

### Affinity of HPr^bs^ for EIN^sc^

The affinity of HPr^bs^ for wild-type EIN^sc^ (Table 1, Fig. 5 A) is six-fold higher than that of HPr^sc^, the natural partner of EIN^sc^ [19]. A similar result was obtained for the formation of the HPr^bs^:EINH186D complex (Table 1, Fig. 5B), where the affinity is four-times larger than that of HPr^sc^:EINH186D complex (∼10 µM; [19]). These results suggest that HPr^bs^ could disrupt the formation of the HPr^sc^:EINH186D complex and therefore, the transfer of phosphate group between both natural partners would be disfavoured.

The binding reactions between HPr^bs^ and wild-type EIN^sc^, or EINH186D, are entropically driven. These results agree with our previous findings describing binding among the un- and phosphorylated species of EIN^sc^ and HPr^sc^ [19]. On the other hand, the differences in the *n*_H_ show diverse mechanisms of binding (Table 1). It is interesting to note that the value of *n*_H_ for HPr^bs^:EIN^sc^ (0.17) is positive, as that previously measured for the EIN^sc^:HPr^sc^ (0.52; [19]). On the other hand, the enthalpy of binding to EINH186D is positive (Table 1), and similar to that of the HPr^sc^:EINH186D [19], suggesting similar binding mechanisms.

### Affinity of HPr^bs^ for EIN^sc^-derived peptides

The affinities of the EINsite peptides for HPr^bs^ were two-fold smaller than that of EIN^sc^:HPr^bs^ complex. This result suggests that there could be other energetically important regions in EIN^sc^ for binding to HPr^bs^, which are not present in any of both peptide sequences. Alternatively, it could indicate a penalty in peptide-binding due to the absence of structure in the isolated fragments [20]. This result disagrees with that reported when EINsite peptides (isolated or in a mixture) were bound to HPr^sc^, where the affinities were similar to that measured in binding to the intact proteins [20]. Therefore, it seems that there are differences in the interactions between both proteins, when shorter polypeptide regions were used. On the other hand, the affinity of an equimolar mixture of both EINsite peptides for HPr^bs^, was similar to those of the complexes involving the isolated peptides (Table 1, Figs. 5 C–E); this similarity suggests that there is not a synergic effect of both peptides on HPr^bs^, similar to what happened with HPr^sc^ [20]. Finally, both EINsite peptide-binding reactions (with both HPr proteins) are entropically driven (Table 1 and [20])), suggesting a large amount of hydrophobic surfaces buried upon binding (as supported by the modelled structure, Fig. 6).

It is interesting to note that the affinities of EINsite peptides for HPr^bs^ were larger than those for HPr^sc^ (as it happened with the intact proteins, see above): the *K*_D_ values were four-times lower than with HPr^sc^ (∼16 µM; [20]). In theory, one should expect a higher affinity between PTS proteins within the same species (unless the interactions are transient). There are four possible explanations for this higher affinity. First, the increase in the affinity could be due to increased functional complementarities. Second, the higher value could be due to conformational differences between HPr^sc^ and HPr^bs^; it could be thought that since close inspection of the modelled structure of the EIN^sc^:HPr^bs^ complex suggests the absence of conformational changes when compared to that of EIN^ec^:HPr^ec^ complex (Fig. 6), unless we have experimental evidence, we cannot rule out fully this second explanation. Next, the higher value could be due to the presence of the self-association equilibrium involving the HPr^bs^; for instance, we have observed that dimerization of the intact EI^sc^ affects its binding to HPr^sc^ [62]. And finally, we have shown that peptides [20], which do not mimic the natural target of EIN^sc^-HPr^sc^ interface (instead, they were obtained by phage display and combinatorial techniques), bound stronger to EIN^sc^ than peptides derived from PTS proteins of the same species. It might be that the HPr^bs^ could have a similar effect on EIN^sc^ than those peptides, and therefore, the reported binding results in this work would be a further step in searching effective disruptors of the PTS protein-protein interactions.

### Biological implications

*B. sphaericus* does not use hexoses or pentoses as the sole carbon source, although it contains *ptsHI* genes encoding HPr and EI proteins belonging to a PTS [10]. These genes are co-transcribed with genes encoding metabolic enzymes related to N-acteylglucosamine catabolism (which can be used as a carbon source by the bacterium). Furthermore, HPr^bs^ shows a low sequence similarity when compared to other phylogenetically-related HPr species, although the two phosphorylation sites (His15 and Ser46) are conserved; moreover, HPr^bs^ is able to bind to EIN from other species (with a larger affinity than the natural partner, as shown this work). All together, and the fact that HPr^bs^ exists in solution as different self-associated species, allow us to hypothesize that HPr^bs^ could be involved in other functions different to that of the PTS, which might be similar or complementary to those carried out by Crh [63] in *B. subtilis*.

## Supporting Information

File S1 Contains: Figure S1: Sequence alignments of (a) HPr^ec^ and HPr^bs^, and (b) EIN^ec^ and EIN^sc^; and Figure S2: The trypsin digestion; are shown in the supplementary information.
(DOC)
