# Supplementary material for: The Histidine-Phosphocarrier Protein of the Phosphoenolpyruvate: Sugar Phosphotransferase System of Bacillus sphaericus Self-Associates
Source: PLoS One. 2013 Jul 26;8(7):e69307. doi: 10.1371/journal.pone.0069307 (PMC3724859; doi:10.1371/journal.pone.0069307)
Supplement: File S1 — Contains: Figure S1: Sequence alignments of (a) HPrec and HPrbs, and (b) EINec and EINsc; and Figure S2: The trypsin digestion; are shown in the supplementary information. (DOC) [file pone.0069307.s001.doc]

**Supplementary Information for the manuscript by Doménech et al.**

FIGURE S1**: EIN and HPr sequence alignments.** (A) Alignment between HPr^bs^ (PTHP_LYSSH) and HPr^ec^ extracted from PDB 3EZA (HPr_3EZA); the sequence similarity was ~ 60%. (B) Alignment between EIN^sc^ (PT1_STRCO) and EIN^ec^ extracted from PDB 3EZA (EIN_3EZA); the sequence similarity was ~55%. The symbols “#” and “-“ correspond to conserved hydrophobic and negatively charged substitutions. The degree of residue conservation is represented by the grey intensity (white, not conserved; dark grey, fully conserved). The secondary structure of the corresponding PDB structure is shown as cylinders (helices) and arrows (β-strands). The figure was obtained with ICM [36].

FIGURE S2. Trypsin digestion of HPr^bs^ (lanes 1-5) and lysozyme (lanes 7-10) carried out at 37 ºC. Incubation times were 0 min (lanes 1 and 7), 5 min (lane 2), 15 min (lanes 3 and 8), 30 min (lanes 4 and 9) and 60 min (lanes 5 and 10). Marker at the lane 6 was the low-molecular range one from Sigma.

Fig. S1 Supplementary Information (Doménech et al.)


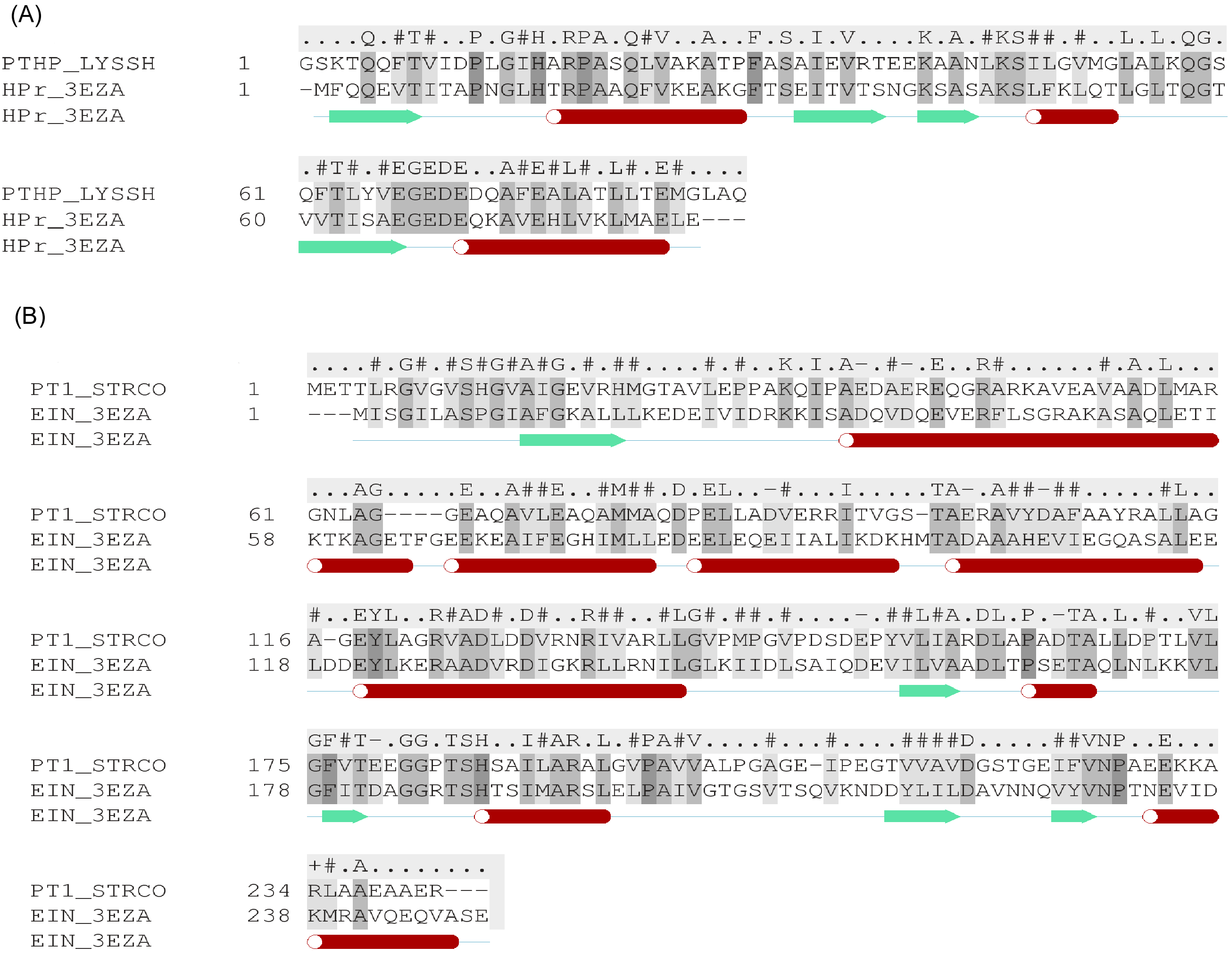


Fig. S2 Supplementary Information (Doménech et al.)


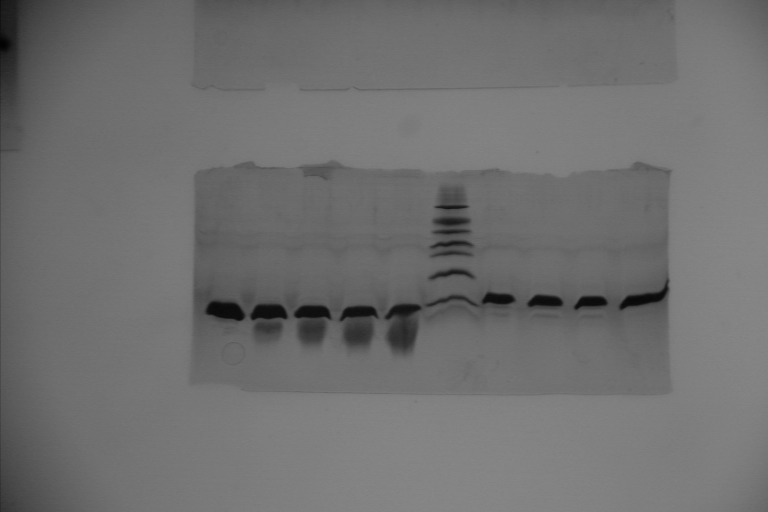


1 2 3 4 5 6 7 8 9 10

14.2 kDa

6.5 kDa
